# Phenotype and function of CXCR5^+^CD45RA^−^CD4^+^ T cells were altered in HBV-related hepatocellular carcinoma and elevated serum CXCL13 predicted better prognosis

**DOI:** 10.18632/oncotarget.6235

**Published:** 2015-10-26

**Authors:** Zhaojun Duan, Jian Gao, Ling Zhang, Hua Liang, Xiangbo Huang, Qiang Xu, Yu Zhang, Tao Shen, Fengmin Lu

**Affiliations:** ^1^ Department of Microbiology and Infectious Disease Center, Peking University Health Science Center, Beijing, China; ^2^ Department of Hepatobiliary and Pancreatic Surgery, Affiliated Tumor Hospital of Zhengzhou University, Zhengzhou, China; ^3^ State Key Laboratory of Infectious Disease Prevention and Control, National Center for AIDS/STD Control and Prevention, Chinese Center for Disease Control and Prevention, Collaborative Innovation Center for Diagnosis and Treatment of Infectious Diseases, Beijing, China; ^4^ Department of Immunology, Peking University Health Science Center, Beijing, China

**Keywords:** T follicular helper cells, CXCL13, HCC, HBV, prognosis

## Abstract

The present study reveals an immunological characterization of circulating and tumor-infiltrating T follicular helper cells (Tfh), namely CXCR5^+^CD45RA^−^CD4^+^ T cells, and their related cytokines in hepatitis B virus-related hepatocellular carcinoma (HCC) patients. In HCC patients, circulating Tfh cells showed a CCR7^+^ and/or ICOS^+^ phenotype with increased Th2-like cells and decreased Th1-like and Th17-like subsets. Although the bulk frequency of circulating Tfh cells was not altered in HCC patients, the frequency of infiltrated CXCR5^+^CD45RA^−^CD4^+^ CD3^+^cells was higher in tumor than in para-tumor tissues, and Th1-like cells were the predominant phenotype. Circulating Tfh cells in HCC patients were defective in the production of IL-21 *in vitro*, which was in accordance with lower IL-21 levels in tumor tissues than in para-tumor tissues. Serum CXCL13 was increased in HCC patients and associated with recurrence-free survival after hepatectomy. This was confirmed in an additional HCC cohort of 111 patients with up to 5 years follow-up. Immunohistochemical staining indicated that the percentage of CXCR5^+^ or CXCL13^+^ cells was higher in poorly differentiated than in well-differentiated tumors. In conclusion, patients with HBV-related HCC showed altered phenotypes and impaired function of Tfh cells or subpopulations. CXCL13 could be a potential biomarker for predicting recurrence in HCC patients after hepatectomy.

## INTRODUCTION

Hepatocellular carcinoma (HCC) is the second most common cause of cancer-related death in the world [1], and more than 50% of liver cancers are associated with hepatitis B virus (HBV) infection [2]. The 5-year survival rate of patients with liver cancer is lower than 15% [3]. The hepatic microenvironment of the host, including the presence of various cytokines and chemokines, is a critical factor affecting the progress and metastasis of HCC. Alterations in the T helper cell (Th1) cytokine profile are associated with metastasis. Specifically, a significant decrease in Th1 cytokines [interleukin (IL)-1A, IL-1B, IL-2, IL-12A, IL-12B, IL-15, interferon (IFN)-γ, and tumor necrosis factor (TNF)] and concomitant increase in Th2 cytokines (IL-4, IL-5, IL-8, and IL-10) are observed in tumor-adjacent noncancerous hepatic tissues in metastatic HCC patients [4, 5].

T follicular helper cells (Tfh) are indispensable for the development of germinal centers (GCs) and the generation of long-term humoral immunity [6–8]. Tfh cells were first discovered in lymphoid tissues with the C-X-C motif chemokine receptor CXCR5 as the canonical marker. However, their memory counterparts in peripheral blood, especially the antigen-specific Tfh-like population, have not been well characterized [9–12]. Circulating Tfh cells typically present as CXCR5^+^CD45RA^−^CD4^+^CD3^+^ and are further classified into three subsets based on CXCR3 and CCR6 expression, namely, Th1-like (CXCR3^+^CCR6^−^), Th2-like (CXCR3^−^CCR6^−^), and Th17-like (CXCR3^−^CCR6^+^) subsets. The Th2-like subset is the primary helper to B cells in the production of immunoglobin [13]. Tfh cells produce interleukin-21 (IL-21), which can activate both innate and adaptive immune responses, and enhance antitumor and antiviral responses [14, 15]. Alterations in Tfh cells are associated with autoimmune and immunodeficiency diseases, and with solid tumors [16, 17]. The frequency of circulating CXCR5^+^ CD4^+^ Tfh cells is increased in chronic hepatitis B patients and positively correlated with alanine aminotransferase (ALT) and aspartate aminotransferase (AST) levels [18, 19]. Circulating CXCR5^+^ CD4^+^ T cells are increased in HBV-related liver cirrhosis and decreased in HBV-related HCC [20].

CXCL13, the only ligand for CXCR5, drives the migration of CXCR5-expressing B cells and Tfh cells to lymphoid tissues and is necessary for the development of secondary lymphoid tissue [21–23]. Circulating CXCL13 levels are increased in many autoimmune diseases and correlated with clinical outcomes [24–26]. CXCL13 is also required for an effective immune response against HBV [27]. Previous work from our group suggested that polymorphisms of CXCL13 are associated with the lack of response to the hepatitis B vaccine [28]. However, the association of CXCL13 with the prognosis of HBV-related HCC remains unclear. In the present study, we examine the frequency and distribution of Tfh cells and Th1-, Th2-, and Th17-like subsets in HBV-related HCC. The secretion of Tfh-related cytokines in patients with HBV-related HCC and the association between alterations in CXCL13 and the progression and prognosis of HCC are evaluated.

## RESULTS

### Tfh subpopulations were redistributed in HCC

**Figure 1:**
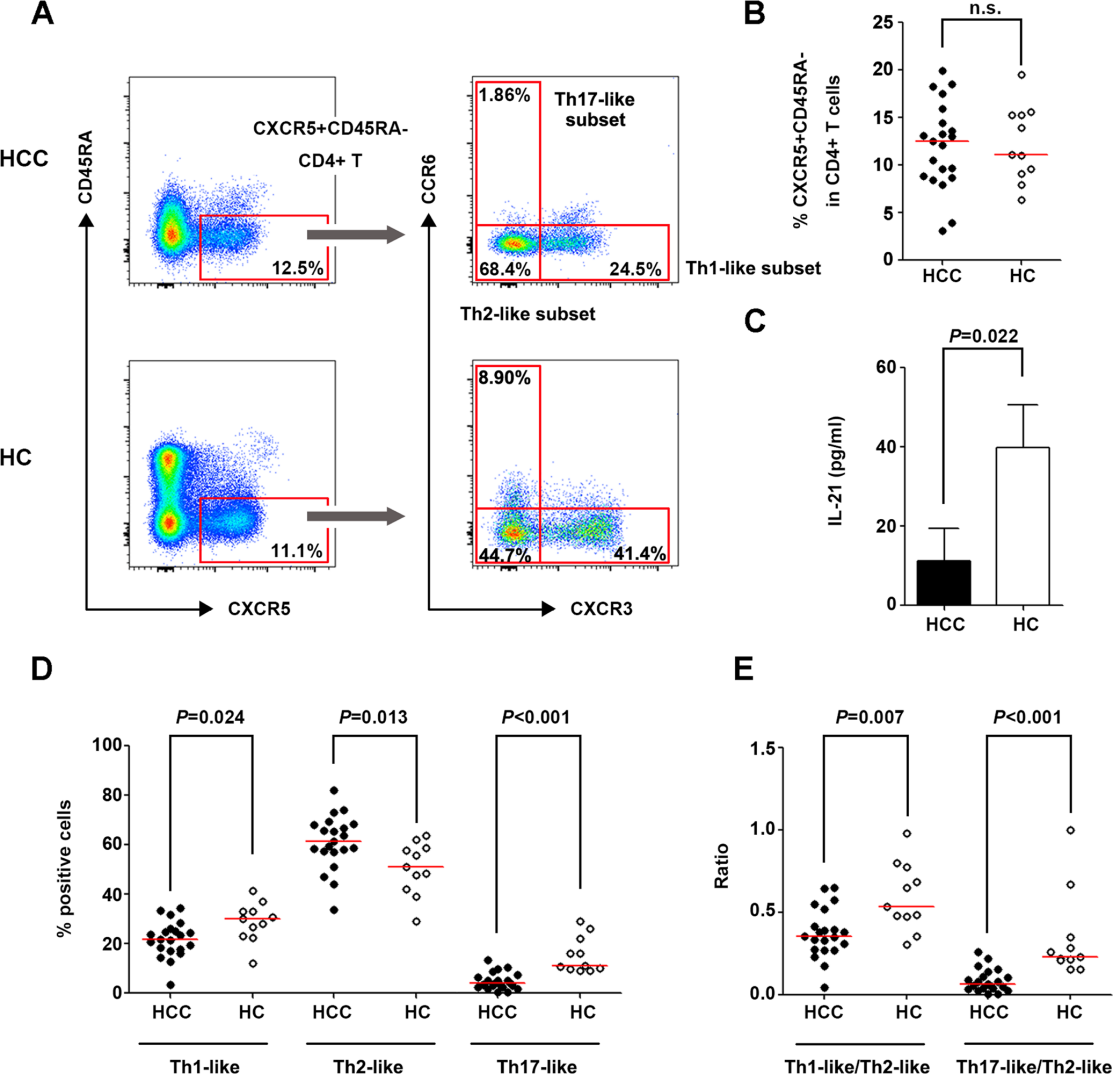
Circulating Tfh cells and cell subsets in patients with HCC and HC (**A**) Gating strategies of circulating Tfh cells and their subsets in HCC and HC. (**B**) Percentage of CXCR5^+^CD45RA^−^cells in CD4^+^ lymphocytes in HCC (*n* = 21) and HC (*n* = 11). (**C**) IL-21 concentration in the co-culture supernatant of purified CXCR5^+^CD45RA^−^CD4^+^CD3^+^ and IgD^+^CD27^−^CD19^+^CD3^−^ cells. (**D**) Distribution of Th1-like, Th2-like, and Th17-like subsets in circulating Tfh cells in HCC and HC. (D) Ratio of Th1-/Th2-like Tfh subsets and Th17-/Th2-like Tfh subsets in HCC and HC. Red lines in (B), (D) and (**E**) show the median values.

The frequencies of circulating Tfh cells and cell subsets were detected in patients with HCC and healthy controls (HC) (Figure 1A). The frequency of CXCR5^+^CD45RA^−^ cells did not differ significantly between the HCC and HC groups (Figure 1B); however, IL-21 concentration was significantly lower in the culture supernatant of sorted CXCR5^+^CD45RA^−^CD4^+^CD3^+^ and IgD^+^CD27^−^CD19^+^CD3^−^ cells from HCC patients than in that from HC (Figure 1C). IgG, IgA, and IgM concentrations in the supernatant could not be tested on day 12 of culture because of extensive cell death in purified cells from HCC patients on days 7 and 8. This suggested that the viability of cells from HCC patients was poorer than that from HC. The Th1-like and Th17-like subsets of circulating Tfh cells were markedly decreased in HCC patients (*P* = 0.024 and *P* < 0.001, respectively, Figure 1D), while the Th2-like subset was increased (*P* = 0.013, Figure 1D) and associated with reduced tumor numbers in HCC patients (*P* = 0.029, Supplementary Table 2). In line with these observations, the ratios of Th1-/Th2-like and Th17-/Th2-like subsets of circulating Tfh cells were remarkably lower in HCC patients than in HC (*P* = 0.007 and *P* < 0.001 respectively, Figure 1E), implying that the tumor environment promoted an increase in the Th2-like subset, which is the primary helper in antibody production, and a decrease in the Th17-like subset, which is the major producer of IL-21.

**Figure 2:**
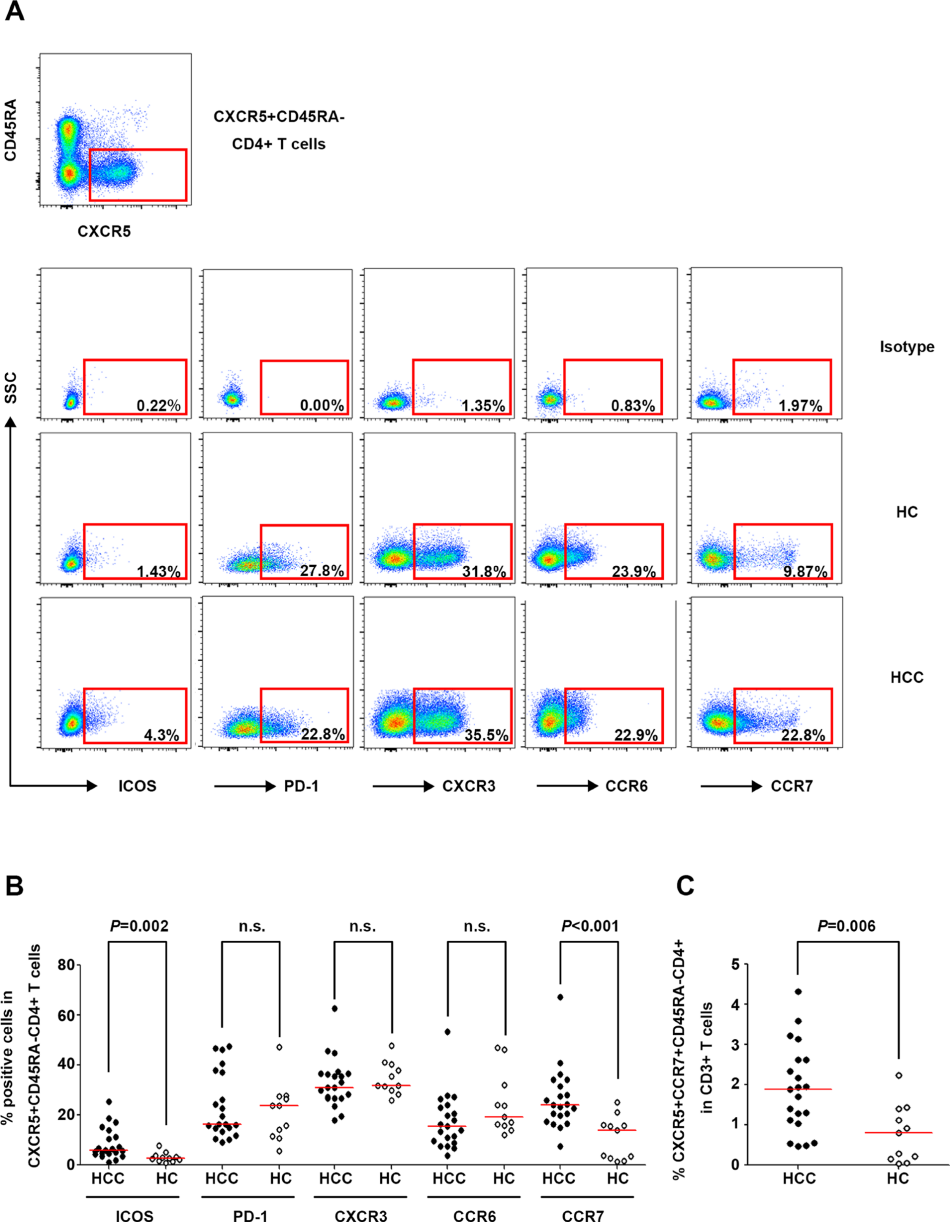
Phenotypic marker expression on circulating CXCR5^+^CD45RA^−^CD4^+^ T cells in HCC and HC (**A**) Expression of the phenotypic markers ICOS, PD-1, CXCR3, CCR6, and CCR7 on circulating Tfh cells in the isotype control (upper panel), a representative HCC patient (middle panel), and a representative HC (lower panel). (**B**) Percentages of ICOS^+^, PD-1^+^, CXCR3^+^, CCR6^+^, and CCR7^+^ cells among circulating CXCR5^+^CD45RA^−^CD4^+^ T cells in HCC (*n* = 21) and HC (*n* = 11). (**C)** Frequencies of circulating CXCR5^+^CCR7^+^CD45RA^−^CD4^+^ cells in HCC and HC. Red lines in (B) and (C) show the median values.

The expressions of ICOS, PD-1, CXCR3, CCR6, and CCR7, which are phenotypic markers for circulating Tfh cells [9, 11, 12, 29], were detected in CXCR5^+^CD45RA^−^CD4^+^ T cells in the HCC and HC groups (Figure 2A). The frequencies of ICOS^+^ and CCR7^+^ CXCR5^+^CD45RA^−^CD4^+^ T cells were higher in HCC patients than in HC (*P* = 0.002 and *P* < 0.001, respectively, Figure 2B), and the percentage of ICOS^+^ Tfh cells was correlated with the incidence of cirrhosis in HCC patients (*P* = 0.039, Supplementary Table 2). Considering that circulating Tfh cells have a central memory phenotype [30, 31], the frequency of circulating central memory Tfh cells (CXCR5^+^CCR7^+^CD45RA^−^CD4^+^) was compared between the HCC and HC groups. The results showed a significantly higher frequency of these cells in HCC patients than in HC (*P* = 0.006, Figure 2C).

Survival analysis showed that the frequency of the Th17-like subset in circulating Tfh cells was positively correlated with recurrence-free survival (log-rank test: *P* = 0.010; Gehan-Breslow-Wilcoxon test: *P* = 0.025, Supplementary Figure 1). Taken together, these results indicated that the distribution of Tfh subpopulations was altered in HCC, and the Th17-like subset was identified as a potential prognostic indicator in HCC.

### Infiltrated CXCR5^+^CD45RA^−^CD4^+^ T cells were increased in tumor tissues compared with para-tumor tissues

**Figure 3:**
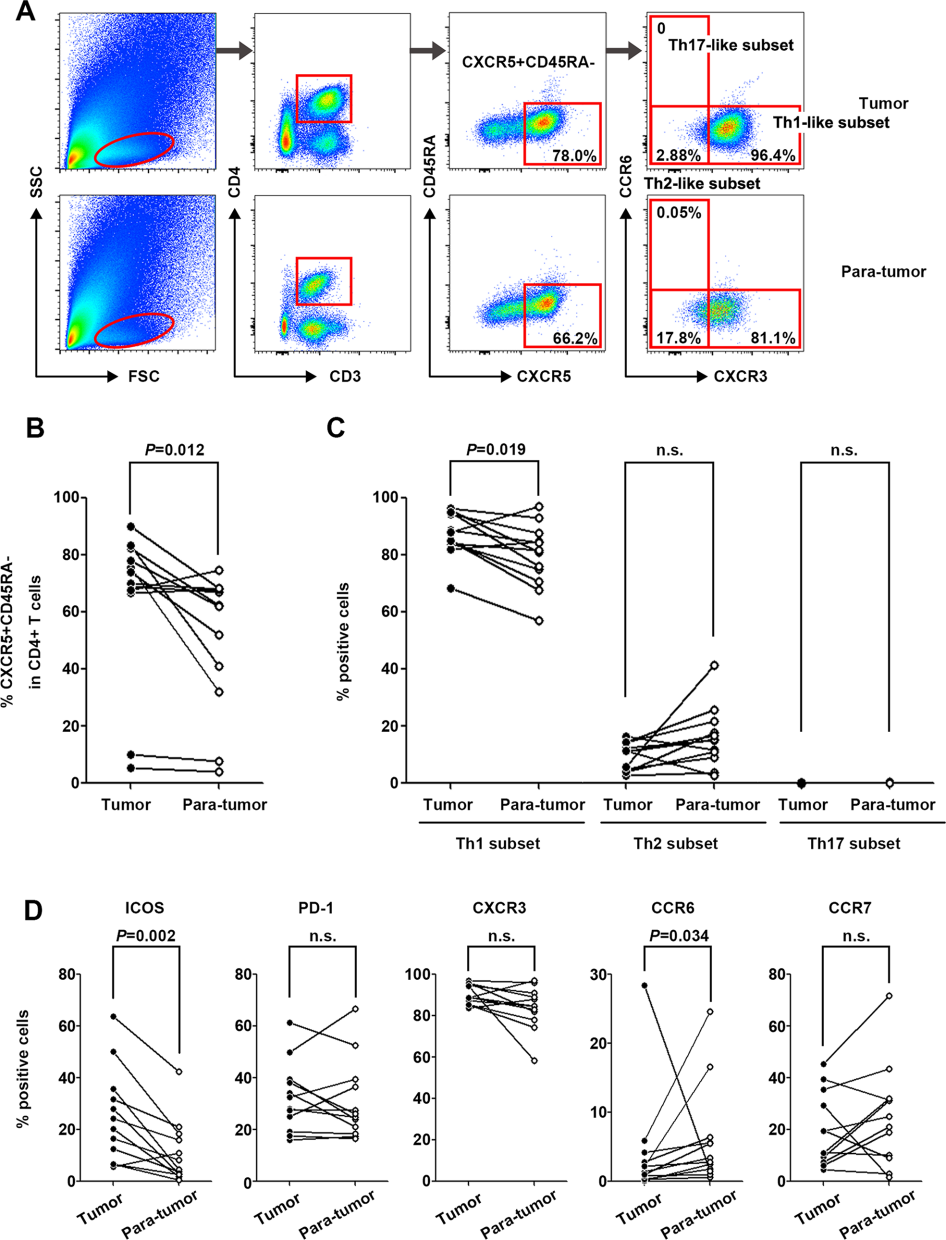
Characterization of tumor-infiltrating CXCR5^+^CD45RA^−^CD4^+^ T cells in tumor and para-tumor tissues (**A**) Typical flow cytometry charts of tumor-infiltrated Tfh cells in tumor (upper panel) and para-tumor (lower panel) tissues. (**B**) Frequencies of CXCR5^+^CD45RA^−^CD4^+^ lymphocytes in tumor and para-tumor tissues (*n* = 12). (**C**) Distribution of Th1-like, Th2-like, and Th17-like tumor-infiltrated CXCR5^+^CD45RA^−^CD4^+^ T cell subsets in tumor and para-tumor tissues. (**D**) Phenotypic marker expression on infiltrated CXCR5^+^CD45RA^−^CD4^+^ T cells in tumor and para-tumor tissues.

Characterization of tumor-infiltrating CXCR5^+^CD45RA^−^CD4^+^ T cells in 12 pairs of matched tumor and para-tumor tissues showed that the population of CXCR5^+^CD45RA^−^ cells among CD4^+^ T cells was higher in tumor tissues than in para-tumor tissues (*P* = 0.012) (Figure 3A and 3B). Unlike the Tfh subsets in peripheral blood, the majority of infiltrated Tfh cells belonged to the Th1-like subset, the frequency of which was significantly higher in tumor tissues than in para-tumor tissues (*P* = 0.019). The frequency of the Th2-like subset was relatively lower and no Th17-like subset was detected in infiltrated Tfh cells (Figure 3C). The percentage of ICOS^+^ Tfh cells was remarkably higher in tumor tissues (*P* = 0.002, Figure 3D) than in para-tumor tissues, showing a similar pattern to that of peripheral blood.

### Serum CXCL13 level was increased in HCC and associated with recurrence-free survival

**Figure 4:**
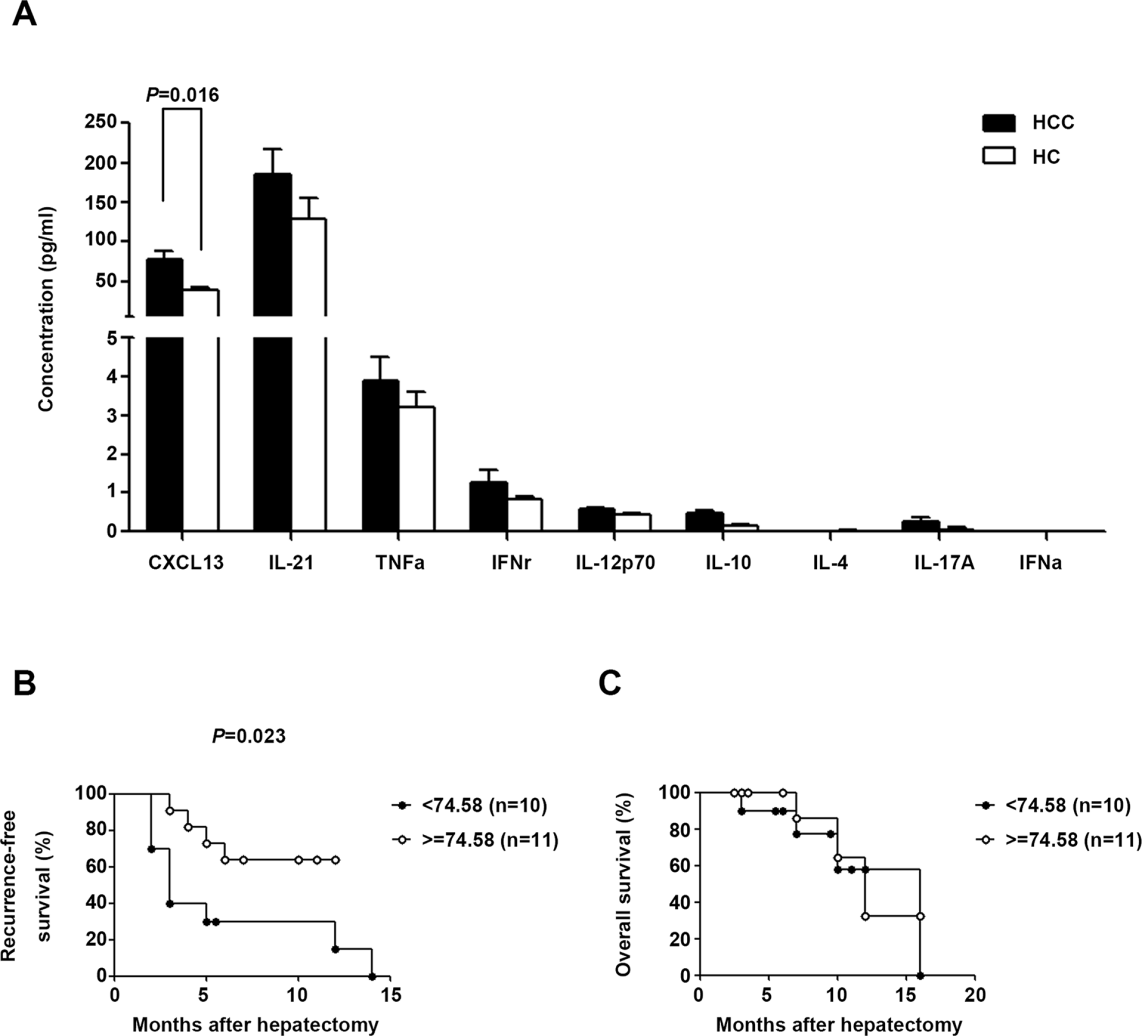
Tfh cell-related serum cytokine production in HCC and HC (**A**) Serum concentrations of cytokines in HCC (*n* = 21) and HC (*n* = 11) subjects. Data are expressed as the mean and SEM. (**B**) and (**C**) Correlation of serum CXCL13 with recurrence-free survival or overall survival. Patients were divided into two groups according to the median concentration of CXCL13.

Given the role of cytokines in the differentiation, migration, and function of Tfh cells, the serum concentrations of nine Tfh-relevant cytokines (CXCL13, IL-21, TNF-α, IFN-γ, IL-12, IL-10, IL-4, IL-17, and IFN-α) were assessed in HCC subjects and HC. CXCL13, the chemokine driving Tfh movement, was significantly up-regulated in HCC patients (*P* = 0.016, Figure 4A) and positively associated with improved recurrence-free survival (log-rank test: *P* = 0.037, Gehan-Breslow-Wilcoxon test: *P* = 0.023, Figure 4B), whereas it was unrelated to overall survival (log-rank test: *P* = 0.696, Gehan-Breslow-Wilcoxon test: *P* = 0.547, Figure 4C)

### CXCL13 was regionally increased in poorly differentiated HCC tumor tissues

**Figure 5:**
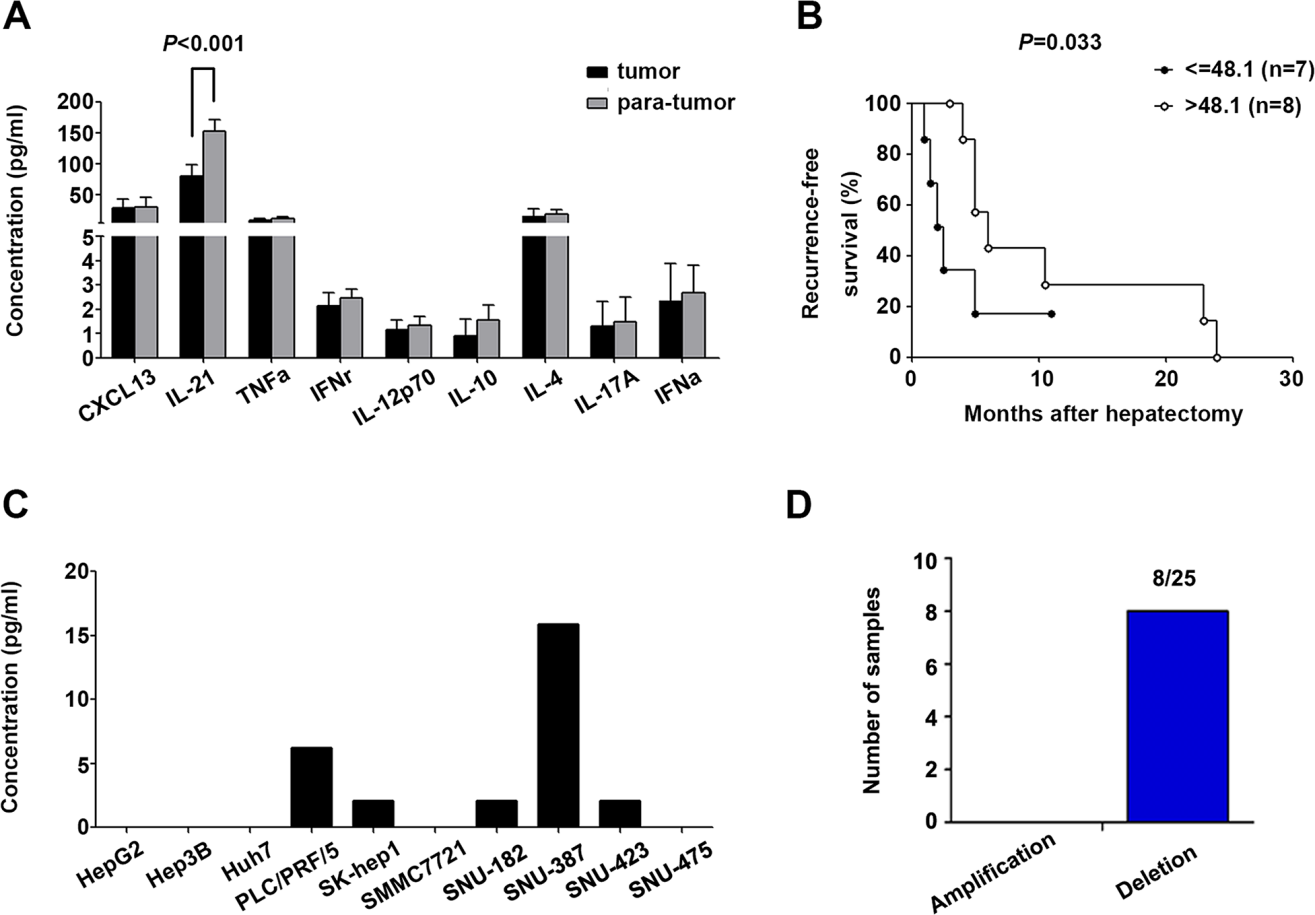
Tfh cell-related cytokine production in HCC tumor tissues and cell lines (**A**) Levels of Tfh-related cytokines in homogenates of 15 paired tumor and para-tumor tissues. (**B**) IL-21 in tumor homogenates was associated with recurrence-free survival. (**C**) Concentration of CXCL13 in the supernatants of ten different HCC cell lines. (**D**) Amplification and deletion status of CXCL13 in 25 paired HCC tumor and para-tumor tissues.

The nine Tfh-relevant cytokines were measured in homogenates of 15 paired tumor and para-tumor tissues from HCC patients. Only IL-21 was higher in tumor homogenates than in para-tumor homogenates (*P* < 0.001, Figure 5A) and positively associated with recurrence-free survival (Gehan-Breslow-Wilcoxon test, *P* = 0.033, Figure 5B). Because plasma CXCL13 levels are correlated with those in the liver in mouse models [27], and our data showed an elevated serum concentration of CXCL13 in HCC patients, the expression status of CXCL13 in tumor tissues was further examined. CXCL13 is secreted by dendritic cells (DC), Tfh cells, and B cells; however, our results showed that CXCL13 was secreted into the culture supernatant of several HCC cell lines (Figure 5C). The results of array-based comparative genome hybridization (aCGH) showed that the *CXCL13* gene was deleted in 8/25 patients (Figure 5D). These results implied that liver cells may have the capacity to produce CXCL13, and the capability of transformed liver cells, such as tumor cells, to produce CXCL13 may be impaired by *CXCL13* gene deletion.

**Figure 6:**
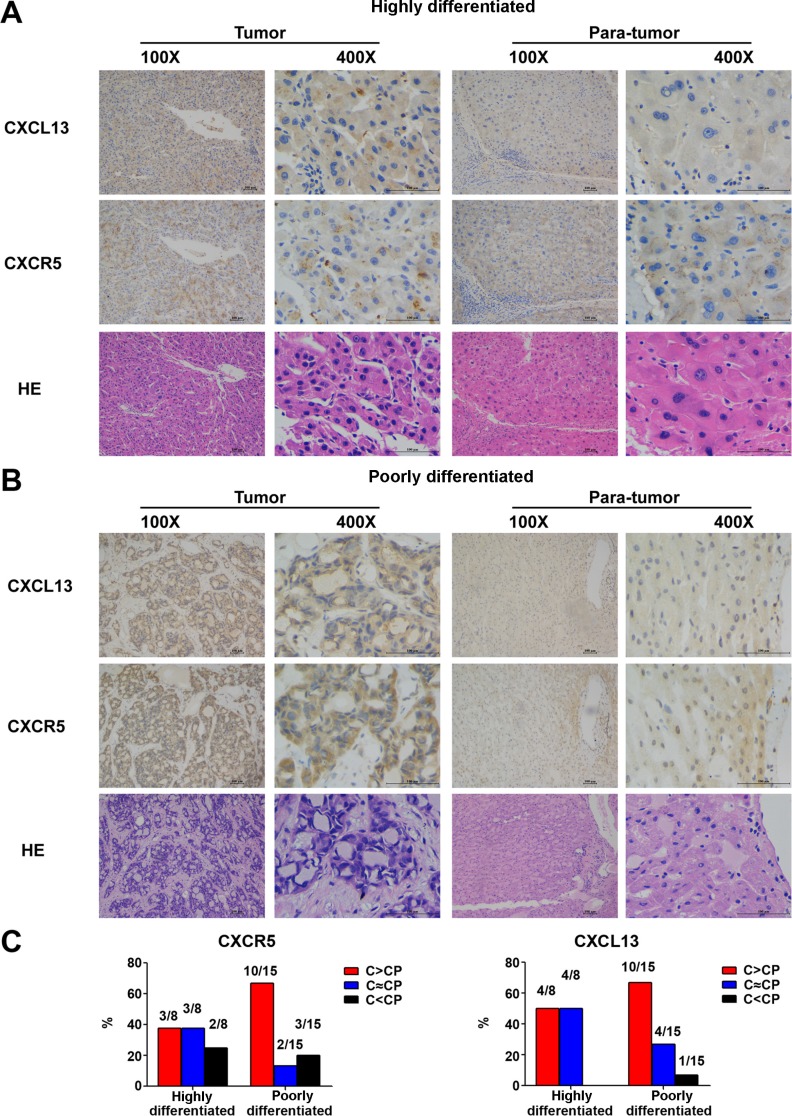
*In situ* expression of CXCL13 and CXCR5 in 23 paired HCC tumor and para-tumor tissues (**A**) Representative images of CXCL13 and CXCR5 immunohistochemistry staining in highly differentiated tumor and para-tumor tissues. (**B**) Representative images of CXCL13 and CXCR5 immunohistochemistry staining in poorly differentiated tumor and para-tumor tissues. (**C**) Comparison of CXCL13 and CXCR5 between tumor and para-tumor tissues. C and CP in the figure stand for tumor and para-tumor tissues, respectively.

Measurement of *in situ* CXCL13 expression by immunohistochemistry (IHC) showed that CXCL13 was expressed in a clustering manner in tumors (Figure 6A and 6B). Samples were then grouped according to the degree of tumor differentiation. In the poorly differentiated group, both CXCL13 and CXCR5 were remarkably higher in tumor tissues (Figure 6C), indicating that CXCL13 was regionally increased in poorly differentiated HCC tumor tissues.

### Serum CXCL13 predicted the prognosis of HCC

**Figure 7:**
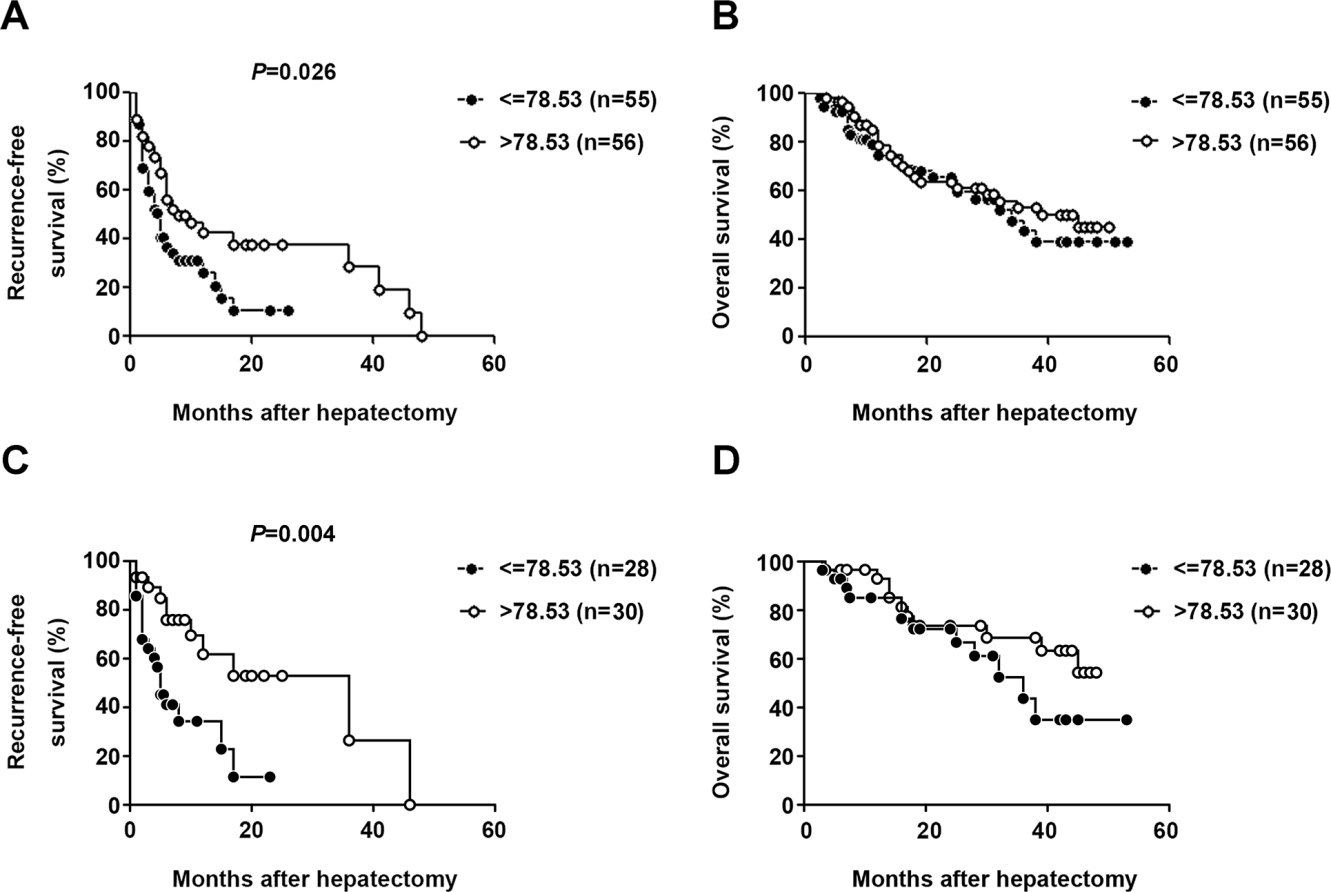
Elevated serum CXCL13 was correlated with recurrence-free survival but not overall survival Association between serum CXCL13 and recurrence-free survival (**A**) or overall survival (**B**) Patients were divided into two groups according to the median concentration of CXCL13. Association between serum CXCL13 and recurrence-free survival (**C**) or overall survival (**D**) especially in HCC patients with AFP lower than 200ng/ml. Patients were divided into two groups according to the same cutoff value of CXCL13 in (A) and (B).

To confirm the role of serum CXCL13 as a predictor of recurrence-free survival, the serum concentration of CXCL13 was measured in 111 HCC patients with detailed 5 years follow-up information. CXCL13 level (median value: 78.53 pg/ml) was highly correlated with recurrence-free survival, but not overall survival, in HCC patients (log-rank test: *P* = 0.011, Gehan-Breslow-Wilcoxon test: *P* = 0.026, (Figure 7A and 7B). In the univariate analysis, in addition to serum CXCL13, serum AFP and γ-GT levels were associated with recurrence-free survival (CXCL13: *P* = 0.011, AFP: *P* = 0.013, γ-GT: *P* = 0.004, Table 1). Since a specific serum AFP cutoff value associated with the prognosis of HCC patients after surgical treatment has not been established, the trend in present study that high level of AFP predicted worse prognosis was in accordance with previous publications [32–36]. To further evaluate the role of serum CXCL13 in the prediction of recurrence-free survival of HBV-related HCC patients, survival analysis were performed especially in those with a lower AFP level (≤200 ng/ml). The results showed significant correlation still existed between serum CXCL13 and recurrence-free survival (Figure 7C), but not over-all survival (Figure 7D). In the multivariate analysis, CXCL13 and γ-GT (CXCL13: *P* = 0.019, γ-GT: *P* = 0.008, Table 1) remained important predictors of recurrence, confirming the role of serum CXCL13 as a prognostic factor in HCC.

**Table 1:** Univariate and multivariate analyses of different parameters associated with HCC using the Kaplan-Meier method and Cox's model

Variables		*N*	Median survival (months)	*P*	
				Univariate analysis	Multivariate analysis
**Gender**	**Male**	90	9.24	0.744	
	**Female**	21	9.25		
**Age**	**< 50**	41	7.89	0.116	
	**≥ 50**	70	10.29		
**CXCL13 (pg/ml)**	**≤ 78.53**	55	8.08	***0.011***	***0.019***
	**> 78.53**	56	10.84		
**Cirrhosis**	**Positive**	96	9.00	0.575	
	**Negative**	15	9.27		
**Tumor number**	**1**	90	9.55	0.233	
	**> 1**	21	8.08		
**Encapsulation**	**Complete**	87	9.51	0.547	
	**Incomplete**	24	8.40		
**AFP (μg/L)**	**≤ 200**	58	11.75	***0.013***	
	**> 200**	53	7.66		
**ALT (U/L)**	**≤ 40**	66	8.78	0.703	
	**> 40**	45	10.09		
**AST (U/L)**	**≤ 40**	59	10.24	0.091	
	**> 40**	52	8.36		
**TBI (μmol/L**	**≤ 17.1**	74	9.38	0.631	
	**> 17.1**	37	9.00		
**γ-GT (U/L)**	**≤ 50**	45	13.90	***0.004***	***0.008***
	**> 50**	66	7.89		
**ALP (U/L)**	**≤ 150**	92	9.17	0.254	
	**> 150**	19	9.60		
**ALB (g/L)**	**≤ 35**	19	10.67	0.557	
	**> 35**	92	9.00		
**Child-Pugh**	**A**	93	8.89	0.945	
	**B**	5	9.00		
**BCLC stage**	**A**	53	9.75	0.280	
	**B and C**	42	8.42		

## DISCUSSION

In the present study, we identified alterations in the distribution of Th1-, Th2-, and Th17-like subsets of circulating Tfh cells in HBV-related HCC patients and showed that the Th17-like subset was correlated with recurrence-free survival. Although the frequency of circulating Tfh cells was not significantly altered in HCC patients, their ability to produce IL-21 was impaired. IL-21 can facilitate effective HBV-specific T and B cell responses, and its therapeutic potential has been tested in clinical trials of combination antineoplastic treatments [37, 38]. The findings of the present study provide further evidence supporting Tfh cells as a potential therapeutic target for the treatment of HCC.

The essential features of circulating Tfh cells in peripheral blood have been described diversely and suggested to be antigen specific. Peripheral Tfh cells show a CCR7^hi^CXCR5^hi^CCR6^hi^PD-1^hi^ phenotype in chronic HIV infections with confirmed B cell helper activity *in vitro* [9]. Circulating PD-1^+^CXCR3^−^CXCR5^+^CD4^+^ memory T cells participate in broadly neutralizing antibody responses in HIV infection and are most related to *bona fide* Tfh cells in GCs [11]. ICOS^+^PD-1^+^CXCR5^+^CD4^+^ T cells are correlated with the functional properties of Tfh cells and play a role in severe systemic lupus erythematosus (SLE) [12]. CCR7^lo^PD-1^hi^CXCR5^+^CD4^+^ T cells are suggested to be the precursors of mature Tfh cells [10]. In the present study, circulating Tfh cells had a predominant central memory phenotype in HCC patients, and the frequency of the ICOS^+^ Tfh subset was increased. ICOS is a co-stimulatory molecule involved in the differentiation of Tfh cells and the production of IL-21 [39, 40]. Although the increase of ICOS indicated that circulating Tfh cells tended to be activated in the tumor environment in our study, the lack of changes in PD-1 expression suggested that Tfh cells were not fully activated actually.

Tumor-infiltrating lymphocytes (TIL) have a predictive value in various tumors [41]. In colorectal cancer, Tfh cells are increased in correlation with tumor progression, whereas other T cells are decreased [16]. Tfh cells infiltrated in peritumoral tertiary lymphoid structures facilitate antitumor responses and predict breast cancer survival [17]. In the present study, serum CXCL13 concentration was identified as a valuable prognostic marker for HCC. Patients with relatively higher CXCL13 levels tended to live longer without recurrence. CXCL13 has a prognosis predictor role in a number of tumors. As reported, CXCL13 is up-regulated in the lungs and plasma of idiopathic pulmonary fibrosis patients, and the extent of CXCL13 overexpression is correlated with cumulative survival [42]. In colorectal cancer, genomic deletion of *CXCL13* predicts a high risk of relapse [16]. CXCL13 in cerebrospinal fluid is a potential prognostic marker for aseptic meningitis [43]. The mechanism underlying the association of CXCL13 with a favorable prognosis is not clear. Davide *et al*. demonstrated that genes strongly correlated with CXCL13 overlap with other beneficial genes, including Th1-related genes [44]. This suggests that a high serum CXCL13 level predicts a better recurrence-free survival via associated genes and cell types. CXCL13 protein levels may be increased by various factors in different cells, and Tfh cells are not the only cell type regulated by CXCL13. This may explain why the bulk frequency of circulating Tfh cells was not significantly elevated in correlation to serum CXCL13 level in the present study.

The concentration and distribution of chemokines/cytokines, which are determined by tumor-infiltrating leukocytes, define the tumor microenvironment and influence tumor growth, underscoring the predictive value of chemokines [37, 45, 46]. CXCL10, CCL5, and CCL2 facilitate the infiltration of T and NK cells and are associated with longer survival in resectable HCC [47]. In the present study, CXCL13 and CXCR5 were up-regulated in tumor tissues, especially in poorly differentiated tumors, suggesting that both CXCL13 and CXCR5 are correlated with HCC and their up- regulation were probably the results of integrated factors in worse tumor environment. CXCL13 could recruit CXCR5 positive Tfh cells and B cells. These cells are the primary source of IL-21 secretion and antibody production, which might benefit tumor cell elimination. So it is understandable that the increased serum CXCL13 in HCC was related to an improved recurrence-free survival. It has been demonstrated that the CXCL13 is dense in portal tracts or sinusoids of the liver in hepatitis C virus- infected patients with mixed cryoglobulinemia and in primary biliary cirrhosis [48, 49]. However, the portal tracts inside tumors were impaired and incomplete in most HCC patients in the present study, making it difficult to conclude that CXCL13 was accumulated in the portal tracts.

In the present study, circulating ICOS^+^ Tfh cells were associated with cirrhosis status. However, paired comparison showed that the percentage of ICOS^+^ Tfh cells was higher in tumor tissues than in para-tumor tissues, implying that the tumor environment rather than cirrhosis status caused the increase in ICOS^+^ Tfh cells.

In summary, the present study was the first to comprehensively analyze the characteristics of circulating and infiltrating CXCR5^+^CD45RA^−^CD4^+^ T cells in HCC patients. Serum CXCL13 was identified as a potential marker to predict recurrence of HCC after hepatectomy.

## MATERIALS AND METHODS

### Study cohorts

Cohort #1: Twenty-one HCC patients treated at the Affiliated Tumor Hospital of Zhengzhou University were enrolled between December 2012 and May 2013, and followed up until October 2014. HCC patients who were positive for serum hepatitis B surface antigen (HBsAg) were included, whereas anti-HIV or anti-HCV positive patients were excluded. The diagnosis of HCC was made according to the standards of the Union for International Cancer Control (UICC) 2010. All samples used in the study were collected from HCC patients who underwent hepatectomy at the hospital and did not receive other treatments before surgery. Blood samples were collected before hepatectomy and peripheral blood mononuclear cells (PBMCs) were isolated. In addition, 12 paired tumor and para-tumor tissues were obtained during the surgery, and tissue-infiltrated lymphocytes were isolated immediately. Blood samples from 11 gender- and age-paired volunteers were used as healthy controls.

Cohort #2: Serum samples from 111 HBV-associated HCC patients who underwent hepatectomy at the Affiliated Tumor Hospital of Zhengzhou University were collected starting in 2009 and patients were followed up until 2014. Patient status including remission, recurrence, or death was clearly recorded. A total of 15 paired tumor and para-tumor tissue samples were collected during hepatectomy and kept at −80°C for the generation of homogenates, and 23 paired tissue samples were prepared for IHC. The detailed demographic information of the participants from cohorts #1 and #2 are listed in Supplementary Table 1. This study was approved by the Ethics Committee of Peking University Health Science Center. Informed consent was obtained from each participant.

### Sample preparation

Sera were stored at −80°C until use. PBMCs were isolated from heparinized anti-coagulated blood using Histopaque-1077 (Sigma-Aldrich, St Louis, MO, USA) according to the manufacturer's instructions. Tumor and paired para-tumor tissues were obtained during surgical resection, and infiltrated lymphocytes were isolated as previously described [50].

Homogenate preparation: Tumor or para-tumor tissues were weighted and RPMI 1640 medium was added (1 ml/100 mg). Tissues were ground and centrifuged, and the supernatant was collected and filtered through a 0.2 μm filter before use.

### Flow cytometry assay

PBMCs were recovered and stained for 30 min at room temperature with a cocktail of antibodies. Monoclonal eFluor450 conjugated anti-CD3 (UCHT1), phycoerythrin (PE) conjugated anti-CXCR5 (MU5UBEE), and fluorescein isothiocyanate (FITC) conjugated anti-ICOS (ISA-3) antibodies were purchased from eBioscience (San Diego, CA, USA). Monoclonal PE- Cy7 conjugated anti-PD-1 (EH12.1), APC-H7 conjugated anti-CD4 (RPA-T4), Alexa Fluor 700 conjugated anti-CD45RA (HI100), PE-CF594 conjugated anti-CXCR3 (1C6/CXCR3), Alexa Fluor647 conjugated anti-CCR7 (3D12), and PerCP-Cy5.5 conjugated anti-CCR6 (11A9) antibodies were purchased from BD Biosciences and BD Pharmingen^™^ (San Diego, CA, USA). After staining, cell samples were detected by BD LSR Fortessa (BD Biosciences). FlowJo software (TreeStar Inc., San Carlos, CA, USA) was used for data analysis.

For cell sorting, three additional monoclonal antibodies were used, including APC conjugated anti- CD19 (HIB19), FITC conjugated anti-IgD (IA6-2), and PE-CF594 conjugated anti-CD27 (M-T271) (BD Pharmingen^™^). Cells were sorted by BD FACS Aria II SORP (BD Biosciences) and resuspended in RPMI 1640 medium for culture.

### *In vitro* co-culture

CXCR5^+^CD45RA^−^CD4^+^ T cells and IgD^+^CD27^−^CD19^+^CD3^−^ B cells were sorted and co-cultured at a ratio of 1:1 (for both cell types, 2 × 10^4^ cells/well were used for immunoglobulin measurement and 5 × 10^4^ cells/well were used for cytokine detection). Cells were cultured in RPMI 1640 containing 1 μg/ml endotoxin-reduced staphylococcal enterotoxin B (SEB), 10% heat-inactivated fetal bovine serum, 1% penicillin-streptomycin, and 1% glutamine. Culture supernatants were collected for cytokine detection on day 2 and for immunoglobulin measurement on day 12.

### Cytokine detection

The concentrations of CXCL13, TNF-α, IFN-γ, IL-12, IL-10, IL-4, IL-17, and IFN-α in sera and tissue homogenates were detected using ProcartaPlex™ Multiplex Immunoassays (Affymetrix, San Diego, CA, USA) according to the manufacturer's instructions. IL-21 (Cloud-Clone Corp, Houston, TX, USA), and IgG, IgM, and IgA (eBioscience, San Diego, CA, USA) were tested using enzyme-linked immunosorbent assay (ELISA) kits.

### Immunohistochemistry

Hepatic tumor and para-tumor tissues from HCC patients were fixed with formalin and embedded in paraffin. Serial sections were prepared for hematoxylin-eosin staining and IHC staining for CXCL13 and CXCR5. For IHC, sections were incubated with primary antibody (CXCL13: R&D, Minneapolis, MN, USA; CXCR5: Abcam, Cambridge, UK) at 4°C overnight, followed by horseradish peroxidase (HRP) labeled secondary antibodies (rabbit anti-goat for CXCL13, KPL, USA; goat anti-rabbit for CXCR5, DAKO, Denmark).

### Real-time RT-PCR

Total RNA from tumor and para-tumor tissues was extracted using the Trizol reagent (Ambion, Austin, TX, USA). The mRNA level of CXCL13 was determined by real-time RT-PCR using SYBR Green I Master Mix (Roche, IN, USA) and detected using a LightCycler 480 II Real-time PCR Detection System (Roche). The primer sequences for *CXCL13* and the housekeeping gene *CTBP1* were as follows: CXCL13-F: 5′-TCCAAGGTGTTCTGGAGGTC-3′, CXCL13-R: 5′-TGAGGGTCCACACACACAAT-3′, CTBP1-F: 5′-TTCACCGTCAAGCAGATGAGAC-3′, and CTBP1-R: 5′-CTGGCTAAAGCTGAAGGGTTCC-3′.

### aCGH

To examine the mRNA levels of CXCL13 in tumor and para-tumor tissues, the chromosome aberration status of CXCL13 was detected as previously reported [51].

### Statistical analysis

All statistical analyses were performed using SAS 9.1.3 (SAS Institute, Inc., Cary, NC, USA). The frequencies of Tfh cells and cell subsets in the different groups were analyzed by the Mann-Whitney *U* test. Comparisons between paired tumor and para-tumor tissues were made using the Wilcoxon matched-pairs signed rank test. The chi-squared test was applied to analyze the correlation between clinical characteristics and laboratory findings. The log-rank test and Gehan-Breslow-Wilcoxon test were used for survival analyses. Univariate and multivariate analyses of the 15 variables involved in HCC were performed using the Kaplan-Meier method and Cox's model. A two-tailed *P-*value of < 0.05 was considered significant.

## SUPPLEMENTARY MATERIAL TABLES AND FIGURE


