# Supplementary material for: Phenotype and function of CXCR5+CD45RA−CD4+ T cells were altered in HBV-related hepatocellular carcinoma and elevated serum CXCL13 predicted better prognosis
Source: Oncotarget. 2015 Oct 26;6(42):44239–53. doi: 10.18632/oncotarget.6235 (PMC4792554; doi:10.18632/oncotarget.6235)
Supplement: Supplementary file 1 [file oncotarget-06-44239-s001.pdf]

Phenotype and function of $CXCR5^{+}CD45RA^{-}CD4^{+}$ T cells were altered in HBV-related hepatocellular carcinoma and elevated serum CXCL13 predicted better prognosis

Supplementary Materials

Supplemental Table S1: Clinical features of the cohorts in this study

	Cohort #1 (*n* = 21)	Cohort #2 (*n* = 111)
**Age**	54 (29–72)	56 (23–78)
**Gender (M/F)**	13/8	91/20
**HBV DNA (log10 IU/ml)**	4.09 (3.83–5.20)	4.16 (3.63–5.42)
**Cirrhosis (Absent/Present)**	3/18	15/96
**Liver Function index**		
ALT (IU/ml)	39 (14–83)	35 (5–485)
AST (IU/ml)	37 (18–127)	37 (14–339)
ALP (IU/ml)	94 (1.5–261)	106 (33–371)
γ-GT (IU/ml)	80 (14–678)	74 (14–399)
AFP (μg/L)	316.9 (1.34–1211)	65.81 (0.3–1211)
ALB (g/L)	41.7 (24.9–49.7)	39 (21.4–50.2)
TBI (μmol/L)	15.2 (5.3–33.2)	14 (5.3–224.3)
**Blood routine**		
RBC ($\times 10^{12}$/L)	4.13 (3.66–4.9)	4.2 (2.8–5.36)
WBC ($\times 10^{9}$/L)	6.5 (2.04–14.25)	5.4 (2–15.8)
PLT ($\times 10^{9}$/L)	141 (30–364)	151 (22–458)

Data were displayed in the form of median (minimum-maximum) except gender and cirrhosis. ALT: Alanine Aminotransferase; AST: Aspartate Aminotransferase; ALP: Alkaline phosphatae; γ-GT: γ glutamyl transpeptidase; AFP: Alpha-fetoprotein; ALB: Albumin; TBI: Total Bilirubin; RBC: Red blood cell; WBC: White blood cell; PLT: Platelet.

Supplemental Table S2: Association between the frequencies of Tfh sub-populations and clinical features of HCC subjects

Variables	Th1 subset/Tfh (%)		Th2 subset/Tfh (%)		Th17 subset/Tfh (%)		ICOS + /Tfh (%)		Central Memory Tfh	
	Values	*P*	Values	*P*	Values	*P*	Values	*P*	Values	*P*
**Age (years)**		0.550		0.130		0.113		0.418		0.860
≤ 50	24.2 (3.41–34.3)		66.1 (44.1–81.9)		3.12 (0.29–9.7)		5.79 (1.21–11.3)		1.55 (0.54–4.32	
> 50	20.7 (12.8–33.3)		58.4 (33.7–74)		5.16 (1.81–13.3)		6.42 (1.81–25.4)		1.96 (0.47–3.59)	
**Gender**		0.856		0.328		0.690		0.328		0.180
Female	22.5 (16–34.3)		58.9 (47.1–68.1)		4.52 (1.91–8.62)		8.46 (3.44–18.7)		1.29 (0.47–3.23)	
Male	21.5 (3.41–33.3)		65.5 (33.7–81.9)		3.52 (0.29–13.3)		5.55 (1.21–25.4)		1.94 (0.55–4.32)	
**Liver cirrhosis**		0.063		0.079		0.078		*0.039*		0.651
Absent	31.6 (23.8–33.3)		57.1 (51.2–57.5)		6.45 (5.16–13.3)		3.62 (1.81–4.58)		1.03 (0.54–3.59)	
Present	21.1 (3.41–34.3)		64.6 (33.7–81.9)		3.44 (0.29–10.3)		6.57 (1.21–25.4)		1.91 (0.47–4.31)	
**Tumor number**		0.967		*0.029*		0.200		0.592		0.091
1	21.6 (3.41–34.3)		64.6 (44.1–81.9)		3.25 (0.29–13.3)		5.79 (1.21–17)		2.05 (0.47–4.32)	
> 1	23.8 (12.8–31.6)		57.1 (33.7–65.5)		5.16 (3.52–7.26)		6.73 (1.81–25.4)		1.03 (0.49–1.96)	
**Encapsulation**		0.303		0.964		0.560		0.893		0.165
Complete	23.4 (14.3–34.3)		61.3 (44.1–81.9)		3.95 (0.29–13.3)		6.04 (1.21–18.7)		1.94 (0.47–4.32)	
Incomplete	17.6 (3.41–31.6)		63.5 (33.7–73.2)		1.83 (2.35–5.16)		8.13 (1.81–25.4)		1.07 (0.55–1.96)	
**AFP (µg/L)**		0.879		1.000		0.849		0.820		0.649
≤ 200	21.5 (3.41–33.3)		61.3 (33.7–73.2)		3.15 (1.81–13.3)		5.55 (1.81–25.4)		1.40 (0.47–3.59)	
> 200	23.8 (14.3–34.3)		59.4 (44.1–81.9)		3.95 (0.29–9.7)		6.73 (1.21–18.7)		1.88 (0.49–4.32)	
TBI (µmol/L)		0.294		0.405		0.971		0.971		0.492
≤ 17.1	23.4 (12.8–33.3)		61.3 (33.7–69.4)		3.52 (0.54–13.3)		6.04 (1.81–25.4)		1.94 (0.47–4.32)	
> 17.1	19.3 (3.41–34.3)		62.5 (47.1–81.9)		3.92 (0.29–7.26)		7.76 (1.21–18.7)		1.50 (0.49–3.23)	
**ALB (g/L)**		0.651		0.802		0.880		0.175		0.175
≤ 35	19.5 (16.9–24.6)		58.1 (47.1–74.0)		3.95 (2.46–7.26)		17.0 (5.03–18.7)		0.49 (0.47–2.62)	
> 35	22.5 (3.41–34.3)		62.5 (33.7–81.9)		3.71 (0.29–13.3)		5.80 (1.21–25.4)		1.91 (0.54–4.32)	
**ALT (U/L)**		0.538		0.856		0.971		0.800		0.538

≤ 40	21.5 (3.41–34.3)		63.7 (33.7–73.2)		3.52 (1.81–13.3)		6.42 (3.44–25.4)		1.94 (0.54–3.59)	
> 40	24.0 (14.3–31.6)		59.7 (44.1–81.9)		4.55 (0.29–9.7)		5.77 (1.21–18.7)		1.98 (0.47–4.32)	
**AST (U/L)**		0.915		0.972		0.915		0.145		0.859
≤ 40	21.6 (12.8–33.3)		62.5 (33.7–81.9)		4.34 (0.29–13.3)		5.23 (1.21–25.4)		1.79 (0.47–3.59)	
> 40	23.8 (3.41–34.3)		59.4 (44.1–74)		3.89 (0.54–9.7)		7.25 (4.58–18.7)		2.62 (0.54–0.49)	
**ALP (U/L)**		0.190		0.190		0.256		0.163		0.930
≤ 150	23.6 (12.8–33.3)		59.9 (33.7–81.9)		5.16 (0.29–13.3)		5.53 (1.21–25.4)		1.79 (0.47–4.32)	
> 150	16.9 (3.41–34. 3)		68.1 (58.4–74)		3.35 (1.81–3.95)		10.5 (5.03–15.2)		2.16 (1.12–2.62)	
**γ-GT (U/L)**		0.447		0.365		0.137		0.294		0.971
≤ 50	22.5 (12.8–33.3)		59.9 (33.7–69.4)		5.16 (2.35–13.3)		5.23 (1.81–25.4)		1.17 (0.55–3.59)	
> 50	19.5 (3.41–34.3)		65.5 (44.1–81.9)		3.35 (0.29–9.7)		6.73 (1.21–18.7)		1.88 (0.47–4.32)	

Data listed were displayed as median (minimum-maximum)

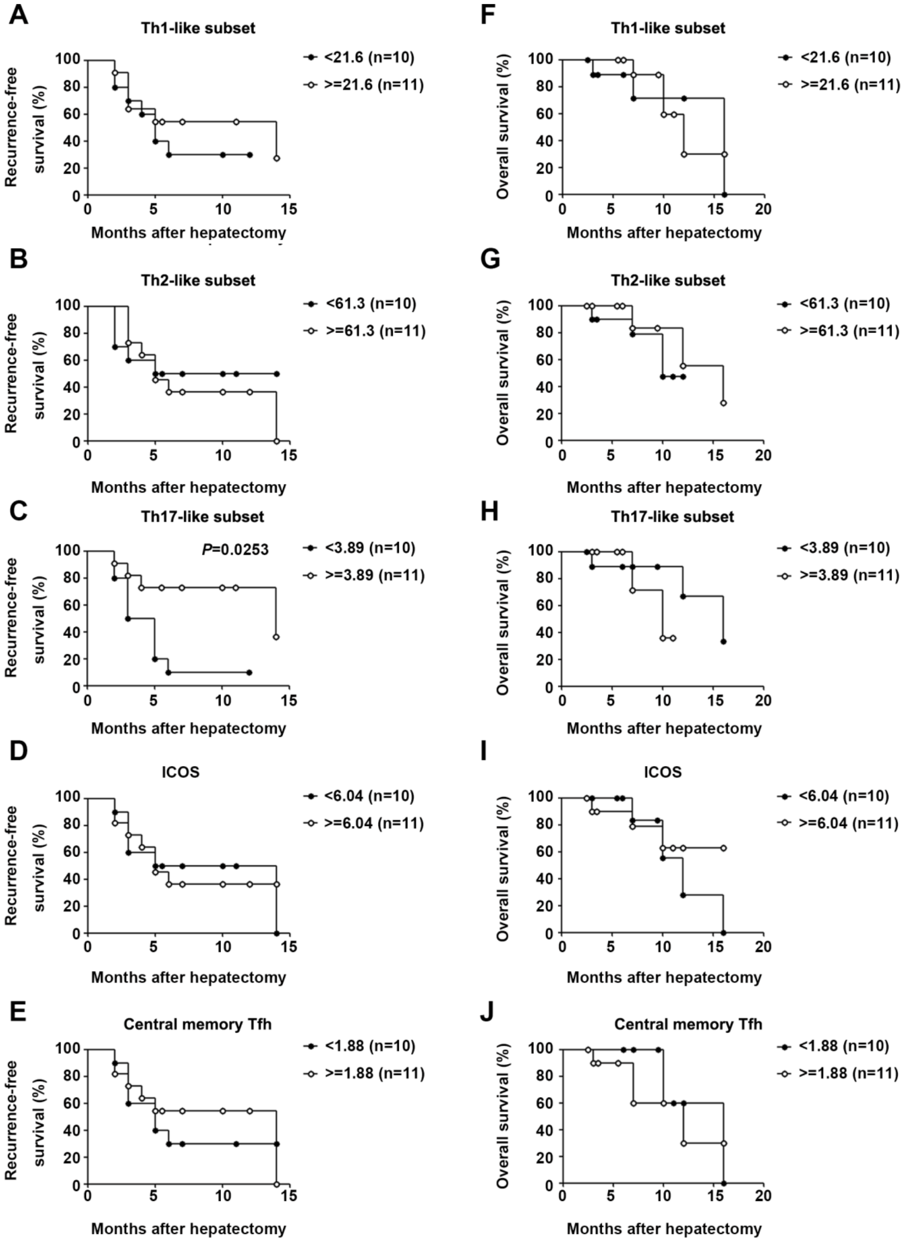

Supplementary Figure S1: Association of Tfh sub-populations with HCC prognosis. (**A**)–(**E**) Association of the frequencies of Th1-like subset, Th2-like subset, Th17-like subset, $ICOS^+$ subset and central memory Tfh cells with recurrence-free survival; (**F**)–(**J**) Association of the frequencies of Th1-like subset, Th2-like subset, Th17-like subset, $ICOS^+$ subset and central memory Tfh cells with overall survival. (Patients were divided into two groups according to median frequencies of each subset).
